# Case series: The role of retinal ischemia in the pathogenesis and clinical manifestation of diabetic retinopathy

**DOI:** 10.1002/ovs2.70090

**Published:** 2026-07-29

**Authors:** Marina Guro, Henrietta Wang, Jack Phu, Kaitlyn A. Sapoznik, Hamza Shah, Maggie L. Zhang, Michael Kalloniatis

**Affiliations:** ^1^ University of Houston College of Optometry Houston Texas USA; ^2^ School of Optometry and Vision Science University of New South Wales Sydney New South Wales Australia; ^3^ Centre for Eye Health Sydney New South Wales Australia; ^4^ School of Medicine (Optometry) Deakin University Melbourne Victoria Australia

## Abstract

**Purpose:**

To highlight retinal ischemia as an important component of diabetic retinopathy (DR) pathogenesis, a significant prognostic factor, and a driver of various DR complications, including neovascularization. We aim to describe the main features of ischemic areas that can be identified through fundoscopy, color fundus photography, optical coherence tomography (OCT), and OCT angiography and provide clinicians with a step‐by‐step approach to identifying prognostically unfavorable markers of DR and accurately staging DR to improve management and prevent severe proliferative DR.

**Case Reports:**

Cases of DR with subtle but significant retinal findings that are not readily identifiable unless a systematic multimodal imaging approach is used. We emphasize the presence of retinal ischemia in each case and show how to identify retinal vascular nonperfusion and its complications.

**Conclusions:**

Advanced stages of DR may lack prominent fundus findings and may be easily misdiagnosed as milder DR without a comprehensive multimodal imaging diagnostic approach.

## Significance

Diabetic retinopathy is a widespread disease and one of the leading causes of legal blindness worldwide. The ability to recognize subtle disease symptoms, including ischemia markers, enables accurate diagnosis and appropriate treatment planning, thereby preventing vision loss from proliferative diabetic retinopathy.

## INTRODUCTION

Diabetes mellitus (DM) is a widespread systemic disease, and diabetic retinopathy (DR), sometimes referred to as diabetic retinal disease, is the most common microvascular complication of DM.^1^, ^2^ The global number of adults suffering from DR in 2020 was estimated at 103.12 million with increased prevalence in North America, the Caribbean, the Middle East and North Africa.^3^ In the United States alone, more than 9 million people had DR in 2021 where it is also the leading cause of blindness in working age adults.^4^ To preserve vision and prevent the progression of DR, it is essential for eye care providers to accurately stage the disease, identify unfavorable prognostic signs, and initiate appropriate modifications for monitoring and treatment.

Developing eye care pathways that connect general practitioners, optometrists, and ophthalmologists improves care for people with ocular diseases.^5^, ^6^ A review of the causes of blindness in England and Wales 1999–2000 with 2009–2010 showed that certified blindness among working age adults was primarily due to inherited retinal dystrophies and not DR. The authors attributed this change to factors such as the introduction of nationwide DR screening programs and improved glycemic control^7^, a conclusion further supported by Thomas et al.^8^ who showed a reduction in DR‐related vision loss despite an increase in the number of patients with DR.

The pathogenesis of DR is complex and involves dysfunction of retinal vascular and neural tissue.^9^, ^10^, ^11^ Hyperglycemia triggers reactive oxygen species formation,^12^ advanced glycation end products (AGE) and receptor for AGEs upregulation,^13^ renin angiotensin system activation,^14^ protein kinase C activation,^15^ and polyol pathway of glucose utilization.^16^ The mechanisms underlying capillary nonperfusion and the formation of ischemic zones are complex. Chronic hyperglycemia leads to activation of the polyol pathway of glucose utilization^16^ and the formation of AGEs. AGEs are the final products of protein glycation, and they can interact with advanced glycation end product receptors on the endothelium of vessels. This interaction induces the production of proinflammatory cytokines, the formation of reactive oxygen species, and the expression of adhesion molecules, leading to inflammation, vascular endothelial dysfunction, and disruption of the blood–retinal barrier.^13^, ^17^ Moreover, AGEs can be formed intracellularly and cause damage to intracellular structures, including mitochondria. Intracellular AGEs disrupt endothelial cell function and can cause apoptosis of endothelial cells and pericytes leading to loss of small capillaries and ischemia. AGEs can also accumulate in basement membrane proteins leading to their thickening. This process interrupts cell–matrix interactions, which further intensifies apoptosis and vascular closure.^13^ Intratissue inflammation is a key pathological mechanism in the development of ischemia. In patients with PDR, increased concentrations of inflammatory cytokines (interleukin‐6 and interleukin‐8) were detected in the vitreous.^18^

Fortunately, retinal imaging tools and measures of retinal function have furthered our understanding of the development and progression of DR and aid in DR detection. With conventional clinical fundus examination and photography, microaneurysms (MAs) are the earliest sign of DR but assessing the retina prior to clinical detection and measuring subclinical alterations that align with the pathophysiology is possible. Retinal function is reduced prior to DR development^19^ and can predict future DR development.^20^ Before clinically detectable DR, the impact of diabetes on blood flow is unclear. In individuals with type 1 diabetes mellitus (T1DM), blood flow was decreased prior to the onset of DR especially with hyperglycemia.^21^ More recent imaging techniques show that blood flow increases prior to the development of DR in individuals with T1DM and type 2 diabetes mellitus (T2DM).^22^ Remodeling of the retinal vessel structure that is not apparent on clinical examination, such as increased wall‐to‐lumen ratio^23^, ^24^ and decreases in vascular mural cell density^25^ in individuals with diabetes with and without retinopathy has also been shown. Additionally, arteriolar narrowing, venular dilatation and arteriovenous nicking can be observed even at the early stages of DR and progress with disease duration. Increase in DR severity is characterized by reduced arteriole‐to‐venule ratio and reflect systematic damage to vascular system.^26^ Further, many aspects of DM retinopathy were associated with arteriole‐to‐venule ratio, central retinal arteriolar equivalent, and retinal venular equivalent.^26^

Under optical coherence tomography angiography (OCTA), perfusion deficits prior to DR onset and the magnitude of capillary nonperfusion increase as DR develops and worsens.^27^ Throughout this manuscript, when we refer to current screening protocols detecting MAs/intraretinal microvascular abnormalities (MAs/IRMAs), this refers to ‘overtly visible DR’, whereas perfusion deficits may not display such overtly visible DR signs.

The ability to assess ischemia is critical because of its role in DR progression. In particular, ischemia is known to upregulate vascular endothelial growth factor (VEGF) and other vascular factors, leading to a breakdown of the blood‐retinal barrier which in turn plays a central role in diabetic macular edema (DME) and neovascularization development.^28^, ^29^, ^30^ However, ischemia is typically subtle, fortunately a combination of retinal imaging techniques, including fundoscopy, color fundus photography (CFP), optical coherence tomography (OCT), and OCTA, can help identify retinal ischemia. Here, we aim to describe the main features of ischemic areas that can be identified through the aforementioned techniques and provide clinicians with a step‐by‐step approach to improve the detection of possible ischemia in patients with DR, as updated classification systems are likely to emerge with the widespread use of retinal imaging modalities able to noninvasively quantify retinal ischemia.^31^, ^32^

Currently in clinical practice, DR progression is commonly assessed using the Early Treatment of Diabetic Retinopathy Study (ETDRS) severity scale^31^, ^33^, ^34^ and the International Clinical Diabetic Retinopathy (ICDR) severity scale.^31^, ^35^, ^36^ Both classifications use similar terminology for the main stages of DR, such as mild, moderate, and severe nonproliferative DR (NPDR), followed by proliferative DR (PDR)—reflecting progression from subtle retinal changes to clear signs of vascular wall damage and dysfunction and neovascularization. The ETDRS classification distinguishes additional sub‐stages for NPDR and PDR, whereas the ICDR classification includes fewer sub‐stages and may be more convenient in routine clinical practice.^31^ Neither of these classifications identifies retinal ischemia as a separate criterion; however, they provide other signs of DR that may serve as indirect indicators of ischemia, such as IRMA, cotton–wool spots, and neovascularization.

### Retinal signs of nonperfusion

The extent and distribution of retinal ischemia can vary. Small foci of ischemia may be present prior to the onset of clinical DR particularly within the deep capillary plexus on OCTA.^37^ However, such subtle changes are difficult to detect with fundoscopy, and OCTA may not be routinely used. In the early stages of NPDR, ischemia is also difficult to detect with fundoscopy, but because other signs are present, it is often inferred that some degree of ischemia is present even without retinal imaging to quantify it. As the disease progresses, an increasing area of the retina becomes involved in the pathological process through the formation and further extension of multiple small ischemic areas. A combination of complete peripheral nonperfusion and diffusely located small capillary dropout areas in the center is observed in the advanced stages of the disease and is typically accompanied by neovascularization.^38^, ^39^

According to the American Academy of Ophthalmology,^34^ slit‐lamp biomicroscopy with a dilated pupil is the preferred method of fundus examination to ensure the proper assessment of both the central and peripheral retina. The peripheral retina, especially the mid‐peripheral regions, commonly has significant areas of nonperfusion that may be missed if a full assessment of the entire retina is not conducted. The peripheral retina should receive close attention during fundoscopy and in assessing CFPs and OCTA scans.^40^, ^41^ Modern imaging modalities, such as ultra‐wide‐field CFP, red‐free FP, OCT, and wide‐field OCTA, enhance the visualization and the documentation of pathological changes of the peripheral retina. Studies show that the use of imaging technologies increases the likelihood of timely referral of at‐risk patients for appropriate treatment.^34^, ^42^

When practitioners evaluate findings from fundoscopy, CFP, and OCTA, the main signs of ischemia that they may find include: cotton–wool spots^43^; areas of loss of the normal vascular pattern‐ghost vessels and capillary dropout^39^, ^44^, ^45^; IRMAs^45^; neural retina edema, including DME^46^, ^47^; and neovascularization.^40^, ^48^

The earliest signs of clinical DR in the fundus are MAs and retinal hemorrhages. Although MAs and retinal hemorrhages are not specific markers of ischemia, their clustering in one area may indicate local nonperfusion (Figure 1A).^49^

**FIGURE 1:**
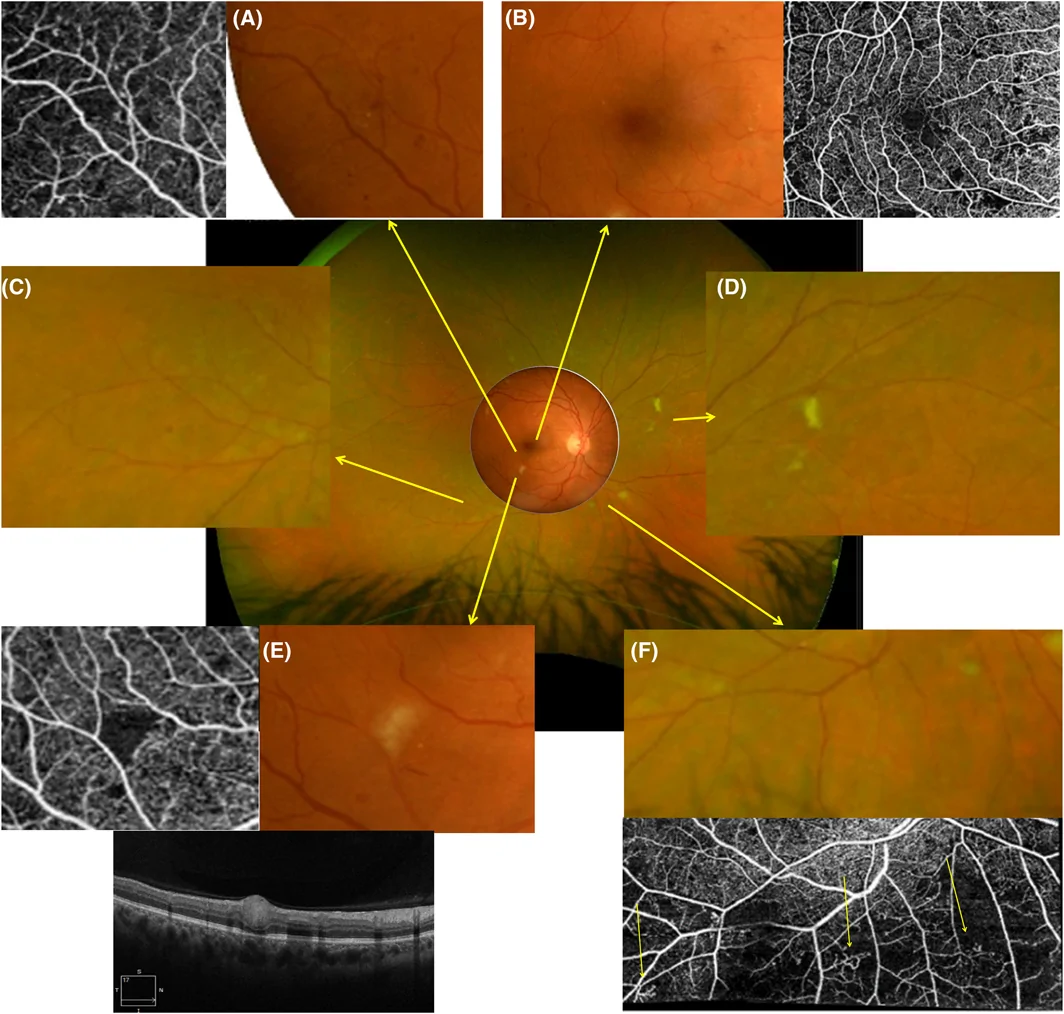
Severe NPDR OD in 43‐year‐old T2DM patient; regular CFP area highlighted in blue shown in the center on top of the wide‐field CFP. (A) Magnified CFP and OCTA of the temporal perimacular region showing capillary dropout and grouped MAs without edema. (B) Magnified CFP and OCTA of the macula showing capillary dropout at the edge of the FAZ and around the fovea. (C) Magnified CFP of the temporal paramacular region showing pale retinal appearance, which is common for affected areas in DR. (D) Magnified CFP of the nasal mid periphery with cotton–wool spots. (E) Magnified CFP, OCTA, and OCT of the inferior macular region showing a cotton–wool spot. (F) Magnified CFP and OCTA with areas of capillary dropout and IRMAs (highlighted by yellow arrows). CFP, color fundus photography; FAZ, foveal avascular zone; IRMAs, intraretinal microvascular abnormalities; MAs, microaneurysms; NPDR, nonproliferative DR; OCTA, optical coherence tomography angiography; OCT, optical coherence tomography.

Cotton–wool spots (Figure 1D,E) are areas of swelling in the retinal nerve fiber layer that appears as white lesions without clear borders and are localized within the neural retina. Anatomically, these lesions represent areas with disrupted orthograde or retrograde axoplasmic transport in ganglion cell axons.^50^ These lesions correspond to areas of nonperfusion on fluorescein angiography (FA), and their size does not fully match the nonperfusion areas on FA. In fact, cotton–wool spots are frequently smaller than the corresponding nonperfused area.^43^, ^51^ Cotton wool spots may appear and disappear depending on the severity of retinal ischemia.^52^, ^53^ However, even after the resolution of cotton–wool spots, retinal function in the affected area does not fully recover due to the formation of a retinal nerve fiber layer defect reflecting axonal loss.^53^, ^54^

Diabetic edema outside and within the macula (DME) can occur with or without ischemia. However, large areas of ischemia may exhibit a degree of edema.^46^ ETDRS introduced the term clinically significant macular edema (CSME) to describe DME located relative to the macula center. In clinical practice, CSME is commonly classified as noncenter‐involved and center‐involved DME. Noncenter‐involved DME refers to retinal thickening that does not involve the central subfield zone at 1 mm in diameter in the foveal region. Center‐involved DME affects the foveal pit and the central subfield zone.^55^ This classification describes the location of the edema but does not give information on whether any signs of ischemia are present or not. OCTA can provide greater insight into the degree of macula perfusion. OCTA‐specific macular sign of ischemia is the enlargement of the foveal avascular zone (FAZ) and small areas of capillary dropout around the FAZ (Figure 1B). These subtle changes may not be accompanied by macular edema. Even so, FAZ enlargement is a strong sign of blood supply deficiency and an unfavorable prognostic factor for DR progression and visual acuity (VA) decline.^38^, ^44^, ^56^ Ischemic areas not localized to the macula may still contribute to DME formation through an overall increase in VEGF production.^47^

Capillary dropout and ghost vessels indicate ischemic areas and are visible on fundoscopy but are even more apparent on OCTA imaging. Areas of ischemic retina may thicken and appear paler during fundoscopy and on CFP compared with the surrounding retina (Figure 1C).^46^ At the same time, a chronic decrease in local capillary blood flow can lead to retinal thinning.^54^

IRMAs are a common finding along the borders of areas of capillary dropout. IRMAs represent tortuous intraretinal vascular segments, which emerge to serve as a passage between arteries and veins (shunt vessels) when capillary circulation is impaired. IRMAs do not show signs of pronounced leakage on FA and do not breach the internal limiting membrane (ILM), distinguishing them from true neovascularization.^45^ They grow at the edges of ischemic areas and appear as dilated, tortuous capillaries.^57^ In most cases, they terminate abruptly and may have dilatations at their tips; more advanced IRMAs can form loops (Figure 1F). They are considered important indicators of DR severity and may be described as intraretinal neovascularization in response to ischemia.^57^ It was confirmed that IRMAs can progress to true neovascularization.^58^, ^59^ IRMAs are usually more apparent under OCTA; however, practitioners can localize them during fundoscopy or in CFP.

Development of neovascularization marks progression from NPDR to PDR. The key distinction between IRMAs and neovascularization is location: IRMAs are located intraretinally and do not encroach the ILM, whereas neovascularization grows between the ILM and the posterior hyaloid membrane (PHM) of the vitreous.^57^ Longitudinal studies have demonstrated that retinal ischemia plays a significant role in DR progression from NPDR to PDR. In the case of high nonperfusion, the rate of progression to PDR stage can be calculated to be roughly 25%–40% at 4 years.^56^, ^60^, ^61^ Therefore, the presence of neovascularization is a strong sign of blood supply deficiency and is characteristic of extensive chronic ischemia.^48^

Table 1 outlines the above points, providing a succinct summary of DR classification. In this case series, we will present clinical information from different imaging modalities. The sequence outlined in the assessment algorithm in Table 2 provides a step‐by‐step approach to diagnosing DR using multimodal imaging.

**TABLE 1:** The relationship between markers of ischemia and the stages of diabetic retinopathy according to ICDR classification.

ETDRS severity scale	ICDR severity scale	Definition
No retinopathy	No apparent retinopathy	No abnormalities
Very mild NPDR	Mild NDPR	MAs only, no specific signs of retinal ischemia
Mild NPDR	Moderate NPDR	– MAs and retinal hemorrhages – hard exudates – venous beading in one quadrant only Ischemia markers: – cotton–wool spots – mild IRMA
Moderate NPDR		
Moderately severe NPDR		
Severe NPDR	Severe NPDR	– Severe retinal hemorrhages in 4 quadrants – Venous beading in at least 2 quadrants Ischemia markers: – Cotton–wool spots – Prominent IRMA in at least 1 quadrant
Very severe NPDR		
Mild PDR	PDR	Ischemia markers: – Neovascularization Elsewhere (NVE) – Neovascularization of the Optic Disc (NVD) – Fibrous proliferation of the Optic Disc (FPD) – Fibrous proliferation elsewhere (FPE) – Vitreous hemorrhages – Preretinal hemorrhages
Moderate PDR		
High‐risk PDR		
Advanced PDR		

*Note:* The ETDRS severity scale is also included to illustrate its correspondence to ICDR stages ^31^, ^33^, ^34^, ^35^, ^36^, ^42^.

Abbreviations: ETDRS, Early Treatment of Diabetic Retinopathy Study; ICDR, International Clinical Diabetic Retinopathy; IRMA, intraretinal microvascular abnormalities; MA, microaneurysms; NDPR, nonproliferative diabetic retinopathy; PDR, proliferative diabetic retinopathy.

**TABLE 2:** A step‐by‐step approach to using multimodal imaging in the diagnosis of diabetic retinopathy.

Type of imaging modality	Clinical use in the diagnosis of DR
1. CFP	Careful evaluation of the CFP helps identify areas of interest (AOI) and reveal signs of ischemia and other pathological changes.
2. Wide‐field CFP	Expands the field of view and identifies AOI with abnormal vessel patterns, grouped MAs or hemorrhages, and IRMAs outside the central vessel arcades.
3. Red‐free FP	Enhances the visibility of vascular structures, including IRMAs and hemorrhages.
4. OCTA	Reveals capillary dropout and/or IRMAs
5. OCT and B‐scans from OCTA	Reveals neural retina thickening or thinning and helps identify abnormal newly formed vessels between ILM and PHM.

Abbreviations: CFP, color fundus photography; DR, diabetic retinopathy; FP, fundus photography; ILM, internal limiting membrane; IRMA, intraretinal microvascular abnormalities; MA, microaneurysms; OCT, optical coherence tomography; OCTA, optical coherence tomography angiography; PHM, posterior hyaloid membrane.

## METHODS

To conduct this study, clinical records from the Center for Eye Health, University of New South Wales in Sydney were reviewed. The Center was an optometry‐led care clinic that provided modern imaging and diagnostic technologies for patients at high risk for various ophthalmic pathologies.^62^ In compliance with the Center's protocols, all reports were reviewed by senior optometrists, with ophthalmology consultations arranged (general ophthalmology or a subspecialty ophthalmologist from the local health district), if needed. The final diagnosis was determined after a thorough assessment of all clinically available results. Subjects with DR diagnoses were selected from the database for this case series. For each case, the authors also reviewed results from different imaging modalities to ensure that all pathological changes were detected. Cases of advanced DR lacking pronounced fundus changes, such as multiple extensive hemorrhages or widespread cystoid macular edema, were selected for this case series. Another inclusion criterion was the presence of subtle ischemic markers, which were listed in Table 1. Due to the absence of distinct fundoscopic signs, the cases could be misdiagnosed without a multimodal imaging approach. An additional inclusion criterion was the absence of previous vitreoretinal interventions. Eyes with a history of retinal laser photocoagulation, intravitreal injections, or vitrectomy were excluded from this case series.

All patients provided written consent for use of their clinical data for research purposes in accordance with the Declaration of Helsinki, and this study was approved by a Biomedical Human Research Ethics Advisory Panel of the University of New South Wales, Australia. Instruments used to collect images for this case series were the fundus retinal camera (Kowa WX 3D nonmydriatic retinal camera, ophthalmic.kowapharma.com/products/retinal‐cameras/nonmyd‐wx3d‐simultaneous‐stereoscopic‐photography‐retinal‐camera‐specs/), Optomap ultra‐widefield with fundus autofluorescence imaging (Optomap Panoramic 200Tx, Optos, optos.com/products/), and OCT (Spectralis HRA2 + OCT, Heidelberg Engineering, heidelbergengineering.com/en‐US/products/spectralis; Cirrus HD‐OCT, Carl Zeiss Meditec, zeiss.com/meditec/en/products/optical‐coherence‐tomography‐devices.html). OCT scans used in this case series include b‐scans through AOI (Spectralis HRA2 + OCT, heidelbergengineering.com/en‐US/products/spectralis; Cirrus HD‐OCT, zeiss.com/meditec/en/products/optical‐coherence‐tomography‐devices.html) and Macular Cube 512 × 128 thickness maps (Cirrus HD‐OCT0, zeiss.com/meditec/en/products/optical‐coherence‐tomography‐devices.html). OCTA scans were acquired using Cirrus HD‐OCT with testing protocols Angiography Analysis 6 × 6 mm, Montage 8 × 8 mm or 12 × 12 mm scans. OCTA assessment was conducted through all available segmentation slabs, and Superficial Vascular Plexus slab was used in figures to highlight ischemic areas.

### Cases

Figure 2A–D represent examples of advanced PDR with extensive neovascularization that may be avoided if prognostic signs indicative of ischemia are detected at the NPDR or early PDR stage. Figure 2A,B show CFPs OU from a 38‐year‐old male with T1DM with advanced proliferative retinopathy. VA was 20/50 OD and 20/30 OS. NVD, NVE, MAs, hemorrhages, and ghost vessels temporal to the macula can be appreciated on the CFP OU (Figure 2A). Note the photocoagulation spots along the edges of the central CFP. Figure 2B is a magnified CFP of the optic nerve head OU with corresponding OCT scans. Note extensive NVD with fibrovascular proliferative membrane. The NVD is located between the ILM and PHM and extends into the vitreous (highlighted by yellow arrows). Figure 2C,D are from a case showing Wolf jaw DR^63^ from a 55‐year‐old male diabetic patient with advanced bilateral PDR and VA of 20/25 OD and 20/30 OS. Only OS is shown. Wide‐field CFP OS (Figure 2C) represents extensive NVD and NVE with fibrovascular membrane formation and vitreous hemorrhage. Note the ghost vessels and the nonperfused areas in the mid‐periphery (highlighted with yellow arrows). Figure 2D is an OCTA scan of the left eye showing a neovascular vitreoretinal membrane that disrupts the inner retinal layers and is attached to the PHM. Note the nonperfused areas in the mid‐periphery (highlighted with a yellow arrow). The macular region is relatively preserved. Regardless of the fact of relatively good VAs in both cases, both patients were at high risk of permanent vision loss due to possible large vitreous hemorrhages and tractional retinal detachment. To prevent the development of such extensive neovascularization sequelae, it is crucial to identify signs of ischemia at the NPDR stage.

**FIGURE 2:**
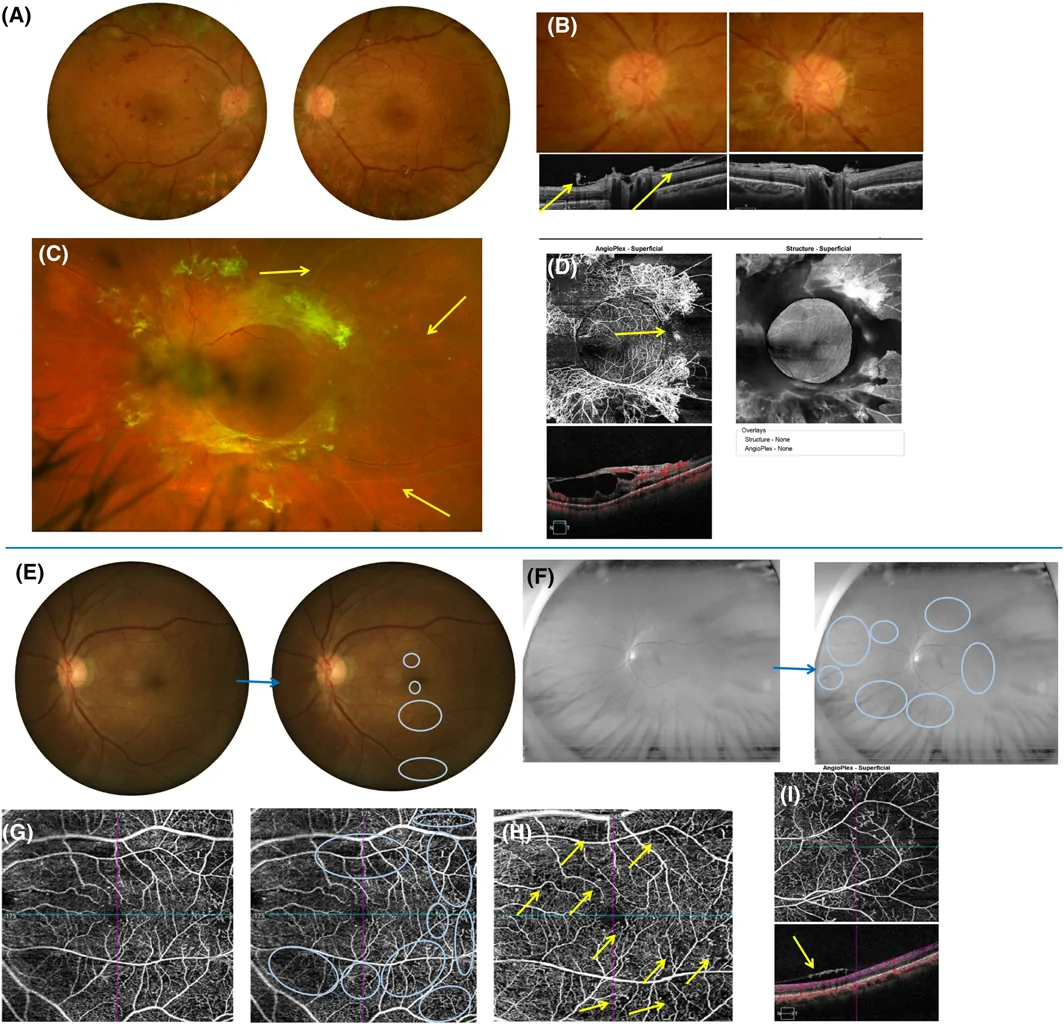
(A, B) 38‐year‐old male with T1DM and VA 20/50 OD and 20/30 OS with advanced proliferative retinopathy. (A) CFP OU, showing NVD, NVE, MAs, hemorrhages, and ghost vessels (temporal to macula). Note the photocoagulation spots along the edges of the photo. (B) Magnified CFP of the optic nerve head OU with corresponding OCT scans. Note extensive NVD with fibrovascular proliferative membrane. NVD is located between ILM and PHM and extends into the vitreous (highlighted by yellow arrows). (C, D) 55‐year‐old male diabetic patient with advanced PDR and VA of 20/25 OD and 20/30 OS. Only OS is shown. (C) Wide‐field CFP OS showing extensive NVD and NVE with fibrovascular membrane formation and vitreous hemorrhage. Note ghost vessels and nonperfused areas at the mid periphery (highlighted by yellow arrows). (D) OCTA OS showing neovascular vitreoretinal membrane that is disruptive to the structure of the inner retinal layers and grows along the PHM. Note nonperfused areas at the mid periphery (highlighted by the yellow arrow). The macular region is relatively preserved. (E–I) A 59‐year‐old male T2DM patient, VA 20/25 OU. (E) Initial CFP OS and the same CFP with highlighted AOI (blue ovals). (F) Wide‐field red‐free FP OS initial and the same FP with highlighted AOI (blue ovals). (G) OCTA OS initial and with highlighted AOI (blue ovals). (H) Magnified OCTA of the macula OS with highlighted capillary dropout (yellow arrows). (I) OCTA of the inferior temporal mid‐periphery region with highlighted neovascularization (yellow arrow). The case shown in panel C, D is from Kalloniatis et al.^63^. AOI, areas of interest; CFP, color fundus photography; ILM, internal limiting membrane; MAs, microaneurysms; NVD, neovascularization of the optic disc; NVE, neovascularization elsewhere; OCTA, optical coherence tomography angiography; OCT, optical coherence tomography; PHM, posterior hyaloid membrane; T2DM, type 2 diabetes mellitus.

#### Case 1

Case 1 of our case series outlines the diagnostic sequence to be used in all subsequent cases (Figure 2E–I). A 59‐year‐old male patient with T2DM for 20 years was referred to the Center for a DR assessment. VA was 20/25 OU; IOP was 13 mmHg OD, 14 mmHg OS. Slit lamp examination showed open angles, mixed nuclear and cortical cataract, and no signs of iris neovascularization. The assessment of the CFP (Figure 2E) revealed several AOIs with MAs and possible IRMAs (highlighted with ovals). Note the absence of hemorrhages, hard exudates, and cotton–wool spots. The major concern at this time was the presence of IRMAs. Wide‐field red‐free FP (Figure 2F) showed additional AOI with IRMAs outside the posterior pole. OCTA (Figure 2G) confirmed capillary dropout and IRMAs (highlighted with ovals). Figure 2H is a magnified OCTA of the macula with highlighted areas of capillary dropout, confirming the presence of ischemia. No macular edema was found. Careful assessment of the OCTA scans revealed an area of neovascularization in the inferior temporal mid‐periphery that was present outside the ILM and was attached to the PHM (Figure 2I). In this case, the diagnostic algorithm (Table 2) helped make a diagnosis of PDR despite the absence of pronounced hemorrhages, exudates, and edema. The patient was referred to an ophthalmologist for further management. If the assessment did not include all the steps outlined in Table 2, a practitioner could miss the early neovascularization detected with OCTA and misclassify this case as mild NPDR. The ability to visualize ischemic areas facilitated the identification of the neovascularization in this case, which can be easily missed if all ischemic areas, including those in the periphery, are not properly assessed for possible vessels extending outside the ILM. If the DR stage is misclassified, the patient is at high risk of quickly progressing to severe PDR. Clinical guidelines recommend that this patient be seen every 4 months; possible panretinal laser photocoagulation may be needed, whereas patients with mild NPDR without macular edema should be seen every 12 months with no active ocular treatment needed.^34^, ^42^

#### Case 2

A 74‐year‐old male with T2DM (HbA1c ∼10%) referred for a glaucoma assessment: IOP 13 mmHg OD and 8 mm Hg OS; VA 20/30 OD, 20/25 OS. He was hospitalized recently due to a stroke and suffered from obstructive sleep apnea, hyperlipidemia, osteoporosis, and anemia. The figures below present a systematic approach, as outlined in Table 2, for making the diagnosis of moderate NPDR in the right eye.

We provide imaging results from the right eye, as the left eye had similar changes. Standard CFP showed no prominent changes, such as hemorrhages or hard exudates. The lack of obvious DR signs could mislead a practitioner into underestimating the severity of the DR in this patient. That is why careful assessment of the retina is very important in every diabetic patient. Meticulous assessment of the CFP revealed areas of fine IRMAs, which are a sign of possible ischemia. These AOIs are highlighted by ovals; the red ovals indicate AOIs that will be described in detail (Figure 3A). Wide‐field red‐free FP identified additional concerning AOIs with IRMAs, cotton–wool spots, and single hemorrhages outside the central vascular arcades (Figure 3B).

**FIGURE 3:**
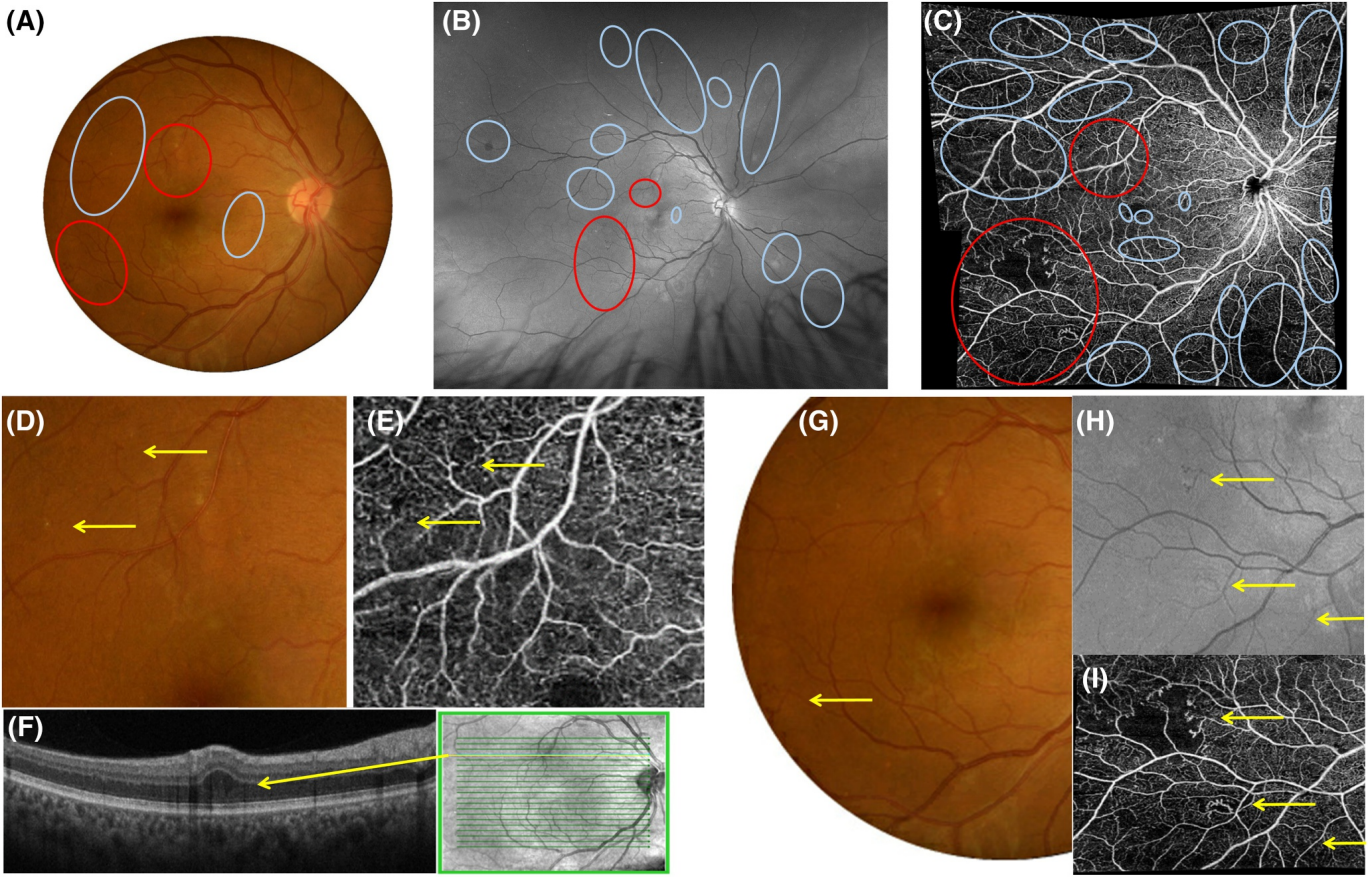
Moderate NPDR with noncenter‐involved DME in a 74‐year‐old male T2DM patient. (A) Color fundus photography of the posterior pole OD with highlighted AOI (ovals). The red ovals indicate AOIs that will be described in more detail. (B) Wide field red‐free FP with highlighted AOI inside and outside the posterior pole. (C) Wide‐field montage OCTA OD with highlighted areas of capillary dropout, MAs, and IRMAs. (D) Magnified CFP view of the superior macular AOI with highlighted IRMAs (yellow arrows). (E) Magnified OCTA through the same AOI with IRMAs and capillary dropout highlighted by the yellow arrows. (F) OCT scan through the same AOI representing local edema. (G) Magnified CFP of the paracentral inferior temporal AOI, IRMA is highlighted by the arrow. (H) Magnified wide‐field red‐free FP of the same AOI with highlighted IRMAs. (I) OCTA of the same AOI with highlighted IRMAs (yellow arrows). AOI, areas of interest; CFP, color fundus photography; DME, diabetic macular edema; IRMAs, intraretinal microvascular abnormalities; MAs, microaneurysms; NPDR, nonproliferative DR; OCTA, optical coherence tomography angiography; OCT, optical coherence tomography; T2DM, type 2 diabetes mellitus.

Wide‐field montage OCTA confirmed capillary dropout in the AOI and revealed even more areas that were ischemic, containing MAs and IRMAs, which were not obvious under the central CFP (Figure 3C). Figure 3D is a magnified CFP of the superior macular AOI highlighted in red. Note the fine IRMAs (yellow arrows). Figure 3E represents an OCTA scan of the same area. There is attenuation of the capillary pattern, MAs, and fine IRMAs at the edges of small ischemic spots (yellow arrows). As IRMAs in this area are very fine, they might be hard to appreciate during fundoscopy or on a standard CFP. In a situation like this, OCTA is the best tool for confirming or refuting the presence of IRMAs. An OCT B‐scan through the AOI showed local edema (Figure 3F), which confirmed an abnormality in this area.

Figure 3G–I are from the paracentral inferior temporal AOI from the same eye. The magnified CFP (Figure 3G) shows IRMA (highlighted with an arrow). Figure 3H is a magnified, wide‐field, red‐free image of the same AOI with highlighted IRMAs. Note that some IRMAs are located outside of the borders of the regular posterior pole CFP and are more apparent under red‐free imaging. OCTA of the same region confirmed the presence of IRMAs along the edges of ischemic areas, which is a common location for this pathology (Figure 3I). No neovascularization was found in the central and peripheral regions. Given the findings, a diagnosis of moderate rather than mild NPDR with noncenter‐involved DME can be made. Clinical guidelines recommend that this patient should be seen every 4–6 months to ensure the signs of DR progression are not missed. A consultation with a retinal ophthalmologist should be obtained in 2–4 weeks.^34^, ^42^

#### Case 3

A 65‐year‐old male with T2DM (HbA1c ∼8%) for over 30 years referred for a DR assessment: IOP 15 mmHg OU; VA 20/20 OD, 20/15 OS. He had no signs of nephropathy or peripheral neuropathy and was only under the care of his family doctor for his diabetes and systemic hypertension. Slit lamp examination showed open angles, no signs of iris neovascularization, and nuclear sclerosis of the lens. Below, we provide a step‐by‐step evaluation of the patient’s right eye.

CFP of the right eye showed areas of cotton–wool spots and hemorrhages along the vascular arcades. One AOI was located at the inferior borderline of the macula (Figure 4A). The lack of hemorrhages and exudates in the posterior pole could lead a practitioner toward a moderate NPDR diagnosis. Wide‐field CFP (Figure 4B) revealed many more AOIs with cotton–wool spots, dot hemorrhages, and prominent IRMAs. Red‐free FP was very helpful in making IRMAs more visible (Figure 4C). Note that IRMAs were accompanied by cotton–wool spots, which are commonly found together as both are signs of ischemic DR changes. Although IRMAs were well formed and easily visible under red‐free FP, they were differentiated from neovascularization because they were confined to the neural retina and did not extend above the ILM on OCT assessment. The paracentral location of the IRMAs is a very common finding in DR, as nonperfused areas are frequently located outside the posterior pole.^40^, ^56^ Macular thickness OCT map confirmed edema inferior to the fovea (Figure 4D). Although the foveal pit had normal contours, the OCT scan through the inferior part of the macula revealed subtle macular edema (Figure 4E). Note that the thickening of the outer nuclear layer is inferior to the fovea, which reaches the foveal pit. The only area of capillary dropout under OCTA was located inferiorly to the fovea (Figure 4F), highlighted with a yellow arrow and corresponded to the local edema detected on OCT. Based on all the findings, including prominent IRMA and macular scans, the diagnosis of severe NPDR with noncenter‐involved DME was made. According to guidelines,^34^, ^42^ this patient should be referred to a retinal specialist for further assessment in 2–4 weeks to determine the need for panretinal photocoagulation and/or anti‐VEGF treatment.

**FIGURE 4:**
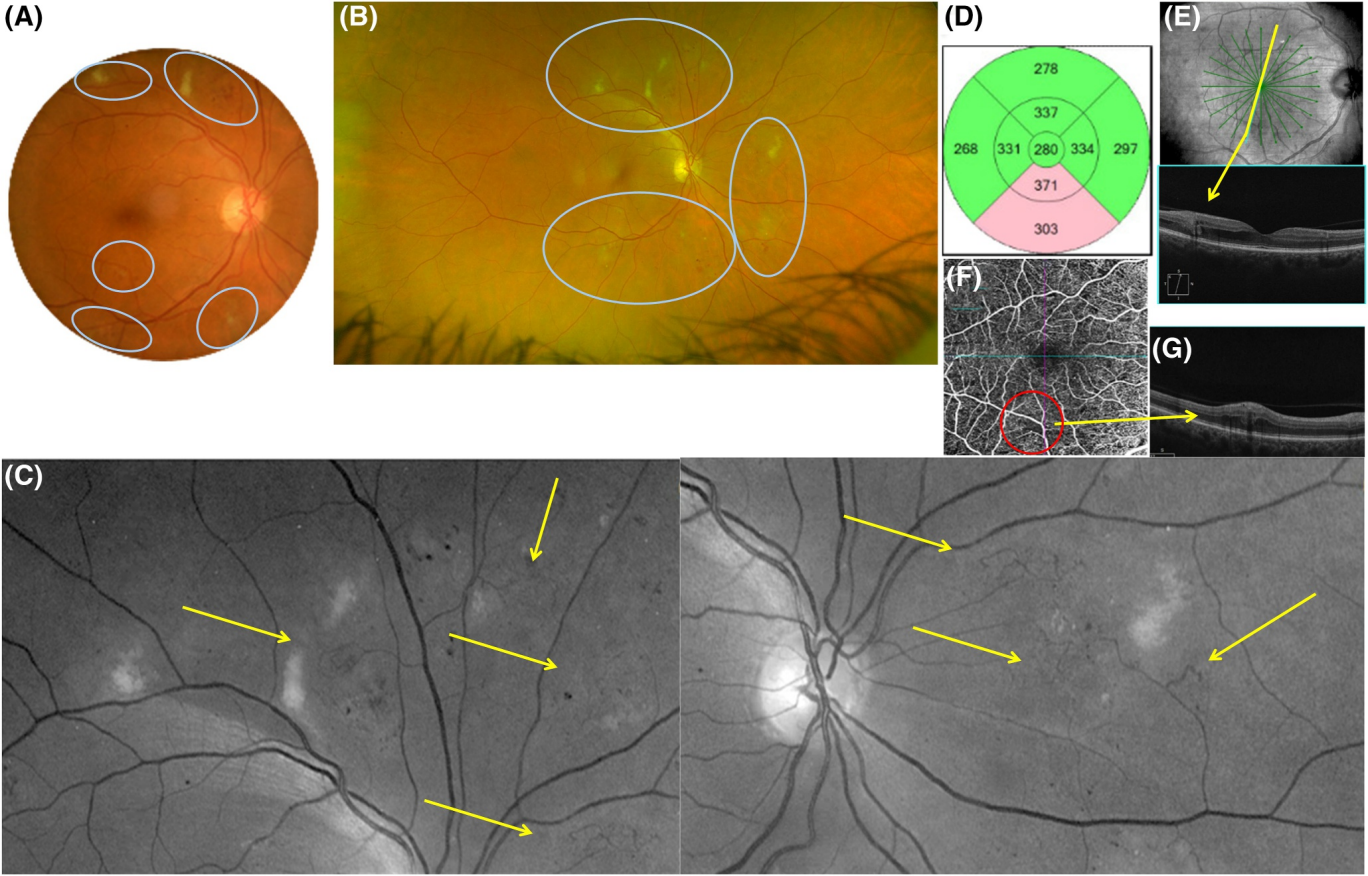
Severe NPDR with noncenter‐involved DME in a 65‐year‐old male T2DM patient. (A) Color fundus photography of the posterior pole OD with highlighted AOI. (B) Magnified wide‐field CFP with highlighted AOI. (C) Magnified wide‐field red‐free FP OD with highlighted areas of IRMA outside the posterior pole. (D) OCT macular thickness map OD. (E) Superior–inferior OCT scan through the fovea OD with highlighted DME adjacent to the area of thickened outer nuclear layer (F) OCTA macular OD with highlighted capillary dropout area. (G) OCT scan OD through the area of edema inferior to the fovea. AOI, areas of interest; CFP, color fundus photography; DME, diabetic macular edema; FP, fundus photography; NPDR, nonproliferative DR; OCTA, optical coherence tomography angiography; OCT, optical coherence tomography; T2DM, type 2 diabetes mellitus.

#### Case 4

A 67‐year‐old male with T2DM (HbA1c ∼13%) referred for a DR assessment: IOP 14 mmHg OD and 16 mmHg OS; VA 20/50 OD, 20/25 OS. The patient had a history of diabetes for over 20 years. Slit lamp examination showed open angles, no signs of iris neovascularization, nor nuclear sclerosis of the lens.

CFP from both eyes showed cotton–wool spots along the vessel arcades, along with dot and blot hemorrhages, hard exudates, and MAs in the macula and along the arcades (Figure 5A). Wide‐field CFP (Figure 5B) and red‐free FP (Figure 5C) detected multiple AOIs with cotton–wool spots and adjacent IRMAs as well as dot and blot hemorrhages outside the posterior pole, nasal, superior, and inferior to the optic nerve. Note that the pattern of the cotton–wool spots and IRMA distribution was the same as in Case 3. OCT through the AOI showed thickening of the neural retina at the nerve fiber layer, ganglion cell layer, and inner plexiform layer in the right eye (Figure 5D) as well as in the left eye (Figure 5E). Macular OCT thickness map revealed center‐involved DME OD (Figure 5F) and an inconsistent decrease in retinal thickness OS (Figure 5G). OCT B‐scan confirmed cystoid DME OD, which aligned with the thickness map (Figure 5H–K). OCT B‐scan OS revealed cysts and thickening within the fovea, which are signs of DME. The reason the thickness map showed the fovea within the normal range is that the overall thickness of the neural retina OS was decreased, whereas edema within the fovea made the foveal retina thicker, bringing the overall thickness to a normal value. This example highlights the importance of careful assessment of retinal structure on OCT scans rather than relying on the device’s ranges. OCTA scans of the macula OU (Figure 5L for OD and Figure 5M for OS) show capillary dropout and MAs at the edges of the FAZ and around the fovea. Summarizing all the findings, the diagnosis of severe NPDR with center‐involved macular edema in both eyes can be made. The patient should be referred to a retinal specialist in 2–4 weeks to determine the need for laser photocoagulation and/or anti‐VEGF treatment.^34^, ^42^

**FIGURE 5:**
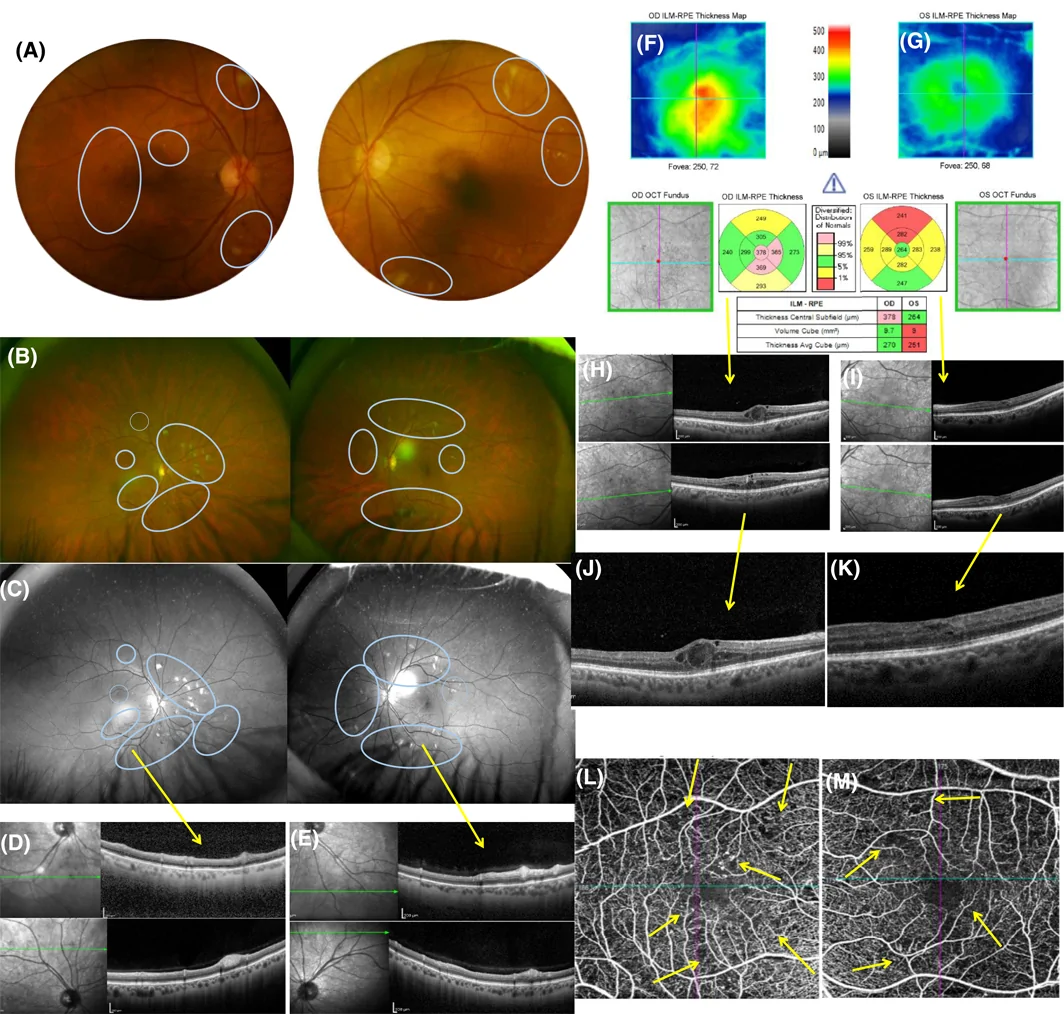
Severe NPDR with center‐involved macular edema OU in a 67‐year‐old male T2DM patient. (A) CFP OU with highlighted AOI. (B) Wide‐field CFP OU with highlighted AOI. (C) Red‐free FP OU with highlighted AOI. (D) OCT scans OD through the AOI with cotton–wool spots. The arrow points to the lesions that correspond to the AOI in panel (C). (E) OCT scans OS through the AOI, showing cotton–wool spots. The arrow points to the lesions that correspond to the AOI in panel (C). (F) OCT thickness map OD. (G) OCT thickness map OS. (H) OCT macular scan OD. (I) OCT macular scan OS. (J) Magnified OCT macular scan OD through the fovea. (K) Magnified OCT macular scan OS through the fovea. (L) OCTA OD with highlighted MAs and capillary dropout. (M) OCTA OS with highlighted MAs and capillary dropout. AOI, areas of interest; CFP, color fundus photography; DME, diabetic macular edema; MAs, microaneurysms; NPDR, nonproliferative DR; OCTA, optical coherence tomography angiography; OCT, optical coherence tomography; T2DM, type 2 diabetes mellitus.

#### Case 5

A 43‐year‐old male with T2DM was referred for a DR assessment (originally described in Kalloniatis et al.^63^). IOPs were 21 mmHg OU; VAs were 20/20‐2 OD, 20/20 OS. He was diagnosed with T2DM, 6 years ago. Slit lamp examination showed open angles, no signs of iris neovascularization, and no lens opacifications. The patient's right eye with severe NPDR is presented in Figure 1.

In the left eye, CFP showed hemorrhages, hard exudates, and cotton–wool spots (Figure 6A). The superior‐temporal AOI had mild IRMAs. These changes were more obvious on the red‐free FP (Figure 6B). When the wide‐field CFP was performed, it revealed additional AOI outside the posterior pole, but no new markers that would change the classification were identified at that step (Figure 6C). Careful analysis of the red‐free FP revealed concerning vessel structures inferior to the optic nerve (Figure 6D, highlighted with the red oval). OCTA confirmed the presence of neovascularization as these vessels were located above the plane of the ILM at the vitreoretinal interface (Figure 6E,F, highlighted with an arrow). Note that capillary dropout is present around the neovascularization. OCTA of the macula also showed focal areas of nonperfusion (Figure 6G). Note the AOI temporal to the fovea, which is highlighted with the red oval. Although there was no edema in the macula (Figure 6H), retinal ischemic perivascular lesions (RIPLs^64^), another sign of ischemia that can be appreciated in DR,^65^ were present in this case. The area of capillary dropout temporal to the fovea showed a decrease in the retinal thickness and wavy appearance (Figure 6I) and corresponded to the capillary dropout visible in the OCTA (Figure 6G highlighted with the red oval). Thinning of the neural retina is a sign of chronic ischemia.^54^ The comprehensive analysis of all findings leads to the diagnosis of PDR OS. This example shows that neovascularization can be subtle and may not be accompanied by obvious signs such as extensive exudates, hemorrhages, or edema. On the contrary, chronic ischemia can lead to decreased retinal thickness accompanied by neovascularization. Per professional guidelines, this case should be evaluated by a retinal specialist to determine whether panretinal laser photocoagulation or anti‐VEGF therapy is needed.^34^, ^42^

**FIGURE 6:**
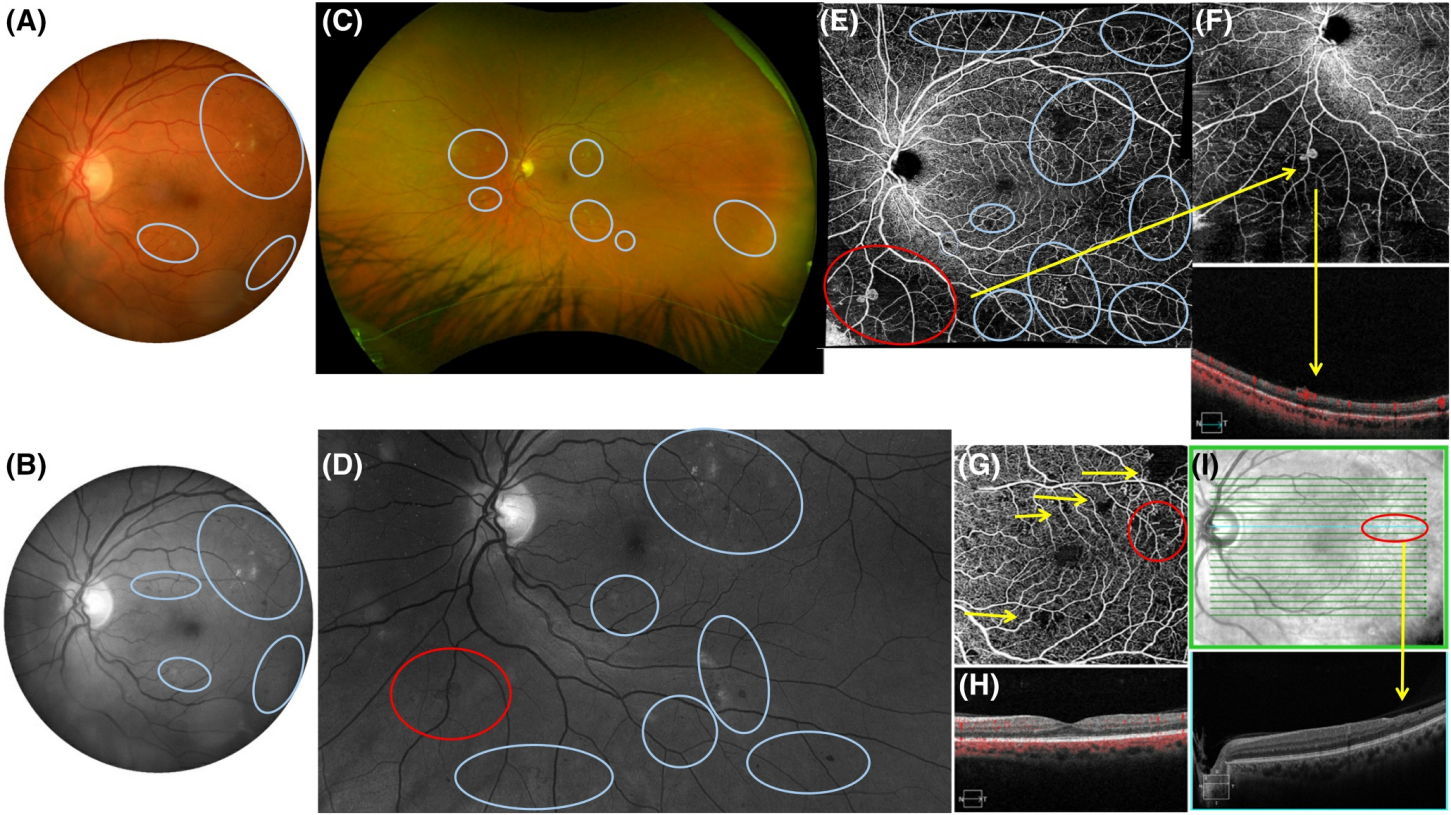
PDR in 43‐year‐old male T2DM patient. (A) CFP OS with highlighted AOI. (B) Red‐free FP OS with highlighted AOI. (C) Wide‐field CFP OS with highlighted AOI. (D) Red‐free wide‐field FP OS. The red oval shows possible neovascularization. (E) Wide‐field montage OCTA OS. (F) OCTA OS with B‐scan through the area of neovascularization. (G) OCTA of the macula OS. (H) OCT B‐scan through the fovea OS. (I) OCT of the macula OS, scan through the superior perifoveal region. This case was previously reported by Kalloniatis et al.^63^. AOI, areas of interest; CFP, color fundus photography; OCTA, optical coherence tomography angiography; OCT, optical coherence tomography; T2DM, type 2 diabetes mellitus.

#### Case 6

A 58‐year‐old female with T2DM (HbA1c ∼7%) was referred for a DR assessment. Entering VAs were OD 20/32, OS 20/25, and IOP was 17 mmHg OU. In addition to T2DM, the patient was under the care of a family practice physician to manage hypertension and hypercholesterolemia. Slit lamp examination showed no signs of anterior segment neovascularization. There were lens opacifications in the right eye and an intraocular lens in the left eye (cataract surgery performed 2 years earlier). For this case, only the data from the left eye is presented.

Wide‐field CFP showed dot and blot hemorrhages, MAs, and cotton–wool spots. Hard exudates were detected in the macula. Although most of the findings on the wide‐field CFP were common in DR, a distinct type of hemorrhage was detected superior to the ONH (Figure 7A; hemorrhage highlighted with an arrow). It was linear, horizontal, and located above the retinal vessels. These signs suggested a preretinal location of the hemorrhage, which, in turn, is a marker of neovascularization.^31^, ^35^, ^42^ Further careful assessment of the CFP revealed areas suspicious for neovascularization along the superior‐nasal vascular arcade and on the ONH (Figure 7B, highlighted with yellow arrows). Red‐free FP made the presumed neovascularization more apparent (Figure 7C). OCTA 12 × 12 mm superficial vascular plexus scan (Figure 7D) showed ischemic areas mostly at the edge of the posterior pole. Note that neovascularization cannot be detected on the OCTA scans that show intraretinal vessels, since neovascularization grows outside the retina between ILM and PHM with or without penetration of PHM. Instead, an OCTA scan of the vitreoretinal interface helped identify abnormal vessels at the surface of the PHM (Figure 7E). An OCT scan through the ONH and the superior nasal vessel arcade confirmed the presence of neovascularization (Figure 7F,G). Despite the normal contour of the foveal pit under OCT (Figure 7I), the macular thickness map identified diffuse DME (Figure 7H). The thickest area was located inferior to the fovea (Figure 7J). Summarizing the findings, a diagnosis of PDR with center‐involved DME should be made for this patient.

**FIGURE 7:**
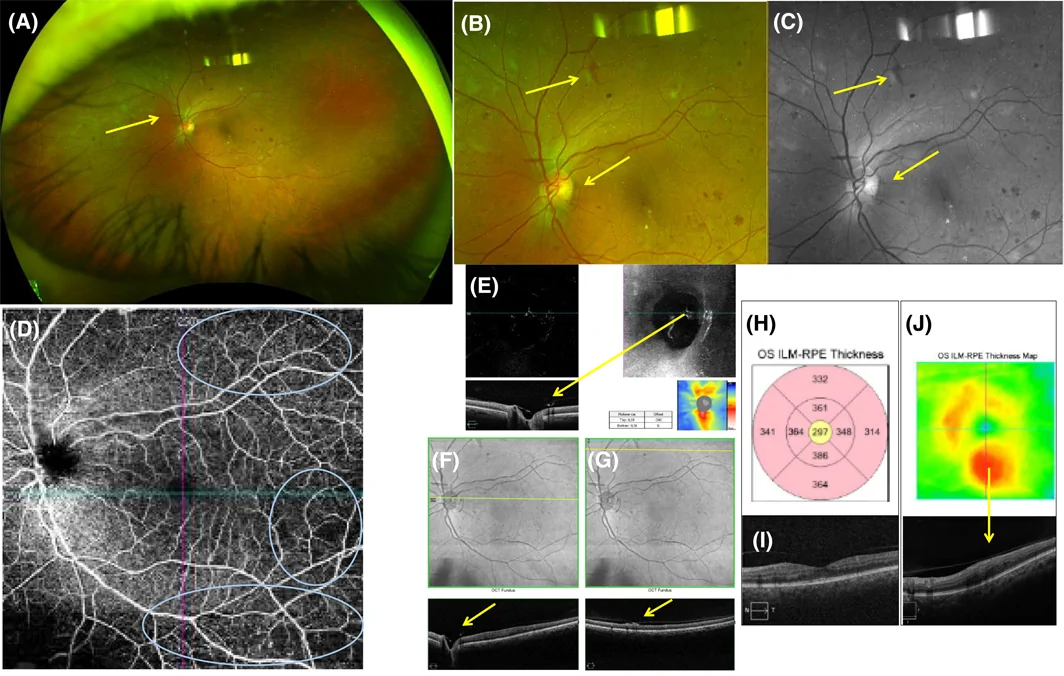
PDR with center‐involved DME in a 58‐year‐old female with T2DM. (A) Wide‐field FP OS, the arrow highlights preretinal hemorrhage. (B) Magnified wide‐field CFP of the AOI around ONH OS, arrows highlight mild neovascularization. (C) Red‐free FP of the same AOI OS, arrows highlight mild neovascularization. (D) 12 × 12 mm OCTA OS (areas of ischemia are highlighted by blue ovals). (E) OCTA of the vitreoretinal interface OS, neovascularization is highlighted by the arrow. (F) OCT scan through the ONH OS, arrow highlights neovascularization. (G) OCT scan through the superior nasal vessel arcade; arrow highlights neovascularization. (H) OCT macular thickness map OS. (I) OCT B‐scan through the fovea OS. (J) OCT map and B‐scan through DME inferior to the macula. AOI, areas of interest; CFP, color fundus photography; DME, diabetic macular edema; MAs, microaneurysms; OCTA, optical coherence tomography angiography; OCT, optical coherence tomography; T2DM, type 2 diabetes mellitus.

This case highlights the importance of subtle changes that mark severe conditions. Sometimes, a small preretinal hemorrhage may be misinterpreted as a retinal hemorrhage. In that case, early neovascularization may be missed. It is important for clinicians to actively look for preretinal hemorrhages during fundoscopy and CFP assessment. The presence of a preretinal hemorrhage of any size should prompt a clinician to investigate its source, which is usually neovascularization in DR. Clinical guidelines recommend that this patient consults a retinal specialist to determine whether panretinal photocoagulation and/or anti‐VEGF treatment are needed.^34^, ^42^

## DISCUSSION

The examples in this case series demonstrate the diversity of DR markers and emphasize that understanding both fundus signs and underlying pathophysiology is crucial. The ability to detect subtle changes, such as capillary dropout and other signs of retinal ischemia, can improve diagnostic accuracy and streamline patient care, thereby improving prognosis.

The areas of nonperfusion and neovascularization are commonly located at mid and far periphery.^40^, ^60^, ^66^ During wide‐field CFP assessment, it is very important to check the retina along the vessel arcades and at the periphery for abnormal vessels, such as IRMA or early neovascularization.^66^ A clinician can start by looking for grouped MAs and cotton‐wool spots, as they indicate areas of nonperfusion. After AOIs with these signs are identified, clinicians can enlarge the picture and precisely check for IRMAs and neovascularization. Red‐free FP modality is particularly useful for this task because it makes blood‐containing structures more visible.^67^

The next step is OCT and OCTA scans assessment. OCT B‐scan is a great tool for assessing retinal thickness and identifying subtle biomarkers such as disorganization of the retinal inner layers (DRIL) in the setting of DME^68^, and retinal ischemic perivascular lesions (RIPLS)^65^, ^69^ can also provide insight into the visual and perfusion status of individuals with DR, although no clear guidelines currently exist regarding the prognostic or monitoring value of such signs. The OCT B‐scan is also a valuable tool for identifying neovascularization, which forms outside the ILM at the vitreoretinal interface and can be easily appreciated on the B‐scan.^70^

OCTA provides information on blood flow within the AOI. It can reveal abnormal vessels, which cannot be seen under CFP or red‐free FP, such as retinal and subretinal neovascularization and IRMA. Wide‐field OCTA is an effective tool for detecting DR, including neovascularization, and has diagnostic accuracy comparable to FA.^71^, ^72^, ^73^ As described above, IRMAs are tortuous intraretinal vascular segments that develop along the margins of a nonperfused area in response to local ischemia. The first description of the histopathology of IRMAs was by Ashton.^74^ The study evaluated postmortem retinal samples from subjects diagnosed with DM. The histopathological findings suggested that initial vessel occlusion occurs on the arteriolar side, leading to new channel formation on the venous side of the intraretinal capillary network. These vessels appear as Y or S‐shaped dilated capillaries that receive circulation from the venules and flow into a vein while remaining connected to obliterated arterioles.^74^ Although IRMAs originate from normal retinal capillaries, they may later evolve into neovascularization, as they can grow and change their structure.^58^, ^59^ For clinicians, it is very important to understand the relationship between IRMA and neovascularization as well as the features that help distinguish them. As we described earlier, the main differences between IRMA and neovascularization are their locations relative to the ILM on OCT B‐scans and the absence of fluorescein leakage on FA. In everyday practice, FA is not routinely used because of its invasive nature; instead, a combination of OCTA and B‐scan modalities makes the location relative to the ILM the primary differential diagnostic feature distinguishing IRMA from neovascularization. However, if a clinician does not have access to OCTA but has FA onsite, FA can still be used to identify nonperfusion and possible neovascularization. OCTA has technical limitations, including the inability to detect leakage from abnormal vessels, variable segmentation across OCTA devices, and imaging artifacts due to eye movement or ocular media opacities.^63^

Although capillary dropout, hemorrhages, exudates, and neovascularization are easily assessable across different diagnostic modalities, evaluation of FAZ enlargement is not that straightforward. The same OCTA scan size should be used for longitudinal comparison of FAZ size across visits. An increase in FAZ with time is a clear sign of DR progression. According to existing findings,^75^, ^76^ there is no significant difference in FAZ size between the right and left eyes in a single person. At the same time, the normal range of FAZ size in healthy subjects is very broad, complicating the initial assessment of baseline size. It is necessary to look for abnormalities in the normal foveal angioarchitecture, such as loss of small capillaries, MAs, and uneven vascular density rather than relying solely on the size value.

Given the complexity of DR pathogenesis and the diversity of clinical presentations, it is essential to use a systematic diagnostic approach, as outlined in Table 2. The imaging modalities described above should be used in combination to ensure that no pathological signs are missed. This approach may help identify very subtle yet clinically significant changes, thereby aiding in accurate diagnosis and determining the appropriate treatment strategy to preserve patients' vision.

## CONCLUSIONS

DR is a complex disease requiring careful evaluation to ensure timely intervention occurs both by the physician dealing with diabetic disease and by ophthalmological intervention through surgical/medical means. Primary care ophthalmic practitioners play a critical role in ensuring cohesive management, which has been shown in major epidemiological trials to reduce the incidence of severe vision loss associated with DR.

## AUTHOR CONTRIBUTIONS

**Marina Guro**: Conceptualization; data curation; writing—original draft; writing—review and editing. **Henrietta Wang**: Data curation; writing—review and editing. **Jack Phu**: Conceptualization; data curation; writing—review and editing. **Kaitlyn A. Sapoznik**: Writing—data curation; writing—review and editing. **Hamza Shah**: Writing—review and editing. **Maggie L. Zhang**: Writing—review and editing. **Michael Kalloniatis**: Conceptualization; data curation; writing—review and editing.

## CONFLICT OF INTEREST STATEMENT

The authors declare no conflicts of interest.
